# The Glycome of Normal and Malignant Plasma Cells

**DOI:** 10.1371/journal.pone.0083719

**Published:** 2013-12-26

**Authors:** Thomas M. Moehler, Anja Seckinger, Dirk Hose, Mindaugas Andrulis, Jèrôme Moreaux, Thomas Hielscher, Martina Willhauck-Fleckenstein, Anette Merling, Uta Bertsch, Anna Jauch, Hartmut Goldschmidt, Bernard Klein, Reinhard Schwartz-Albiez

**Affiliations:** 1 Medizinische Klinik V, Universitätsklinikum Heidelberg, Heidelberg, Germany; 2 Translationale Immunologie, Deutsches Krebsforschungszentrum Heidelberg, Heidelberg, Germany; 3 Nationales Centrum für Tumorerkrankungen, Heidelberg, Germany; 4 Pathologisches Institut, Universität Heidelberg, Heidelberg, Germany; 5 INSERM, U1040, Montpellier, France; 6 Centre Hospitalier Universitaire, Montpellier, Institute of Research in Biotherapy, 3 Montpellier, France; 7 Université Montpellier 1, UFR Médecine, Montpellier, France; 8 Abteilung für Biostatistik, Deutsches Krebsforschungszentrum Heidelberg, Heidelberg, Germany; 9 Institut für Humangenetik, Universität Heidelberg, Heidelberg, Germany; Cordelier Research Center, INSERMU872-Team16, France

## Abstract

The glycome, i.e. the cellular repertoire of glycan structures, contributes to important functions such as adhesion and intercellular communication. Enzymes regulating cellular glycosylation processes are related to the pathogenesis of cancer including multiple myeloma. Here we analyze the transcriptional differences in the glycome of normal (n = 10) and two cohorts of 332 and 345 malignant plasma-cell samples, association with known multiple myeloma subentities as defined by presence of chromosomal aberrations, potential therapeutic targets, and its prognostic impact. We found i) malignant vs. normal plasma cells to show a characteristic glycome-signature. They can ii) be delineated by a lasso-based predictor from normal plasma cells based on this signature. iii) Cytogenetic aberrations lead to distinct glycan-gene expression patterns for t(11;14), t(4;14), hyperdiploidy, 1q21-gain and deletion of 13q14. iv) A 38-gene glycome-signature significantly delineates patients with adverse survival in two independent cohorts of 545 patients treated with high-dose melphalan and autologous stem cell transplantation. v) As single gene, expression of the phosphatidyl-inositol-glycan protein M as part of the targetable glycosyl-phosphatidyl-inositol-anchor-biosynthesis pathway is associated with adverse survival. The prognostically relevant glycome deviation in malignant cells invites novel strategies of therapy for multiple myeloma.

## Introduction

Multiple myeloma is a rarely curable malignant disease of clonal plasma cells which accumulate in the bone marrow causing clinical signs and symptoms related to the displacement of normal hematopoiesis, formation of osteolytic bone lesions, and production of monoclonal protein [1]. Myeloma cells harbor a high median number of chromosomal aberrations [2], [3] and multiple changes in gene expression compared to normal bone marrow plasma cells. This molecular heterogeneity is thought to transmit into very different survival times ranging from a few month to 15 or more years [4], with a median survival after conventional treatments of 3–4 years and 5–9 years after high-dose treatment followed by autologous stem-cell transplantation [5]–[7].

One hallmark of normal and malignant plasma cells is a bidirectional communication with the bone marrow microenvironment, especially processes like bone turnover and angiogenesis [8]–[10]. Although it is well known that the glycan moiety of glycoproteins, proteoglycans and glycosphingolipids has an important impact on the function of the intact macromolecule, both under physiological or malignant conditions, only scarce information on the glycosylation in multiple myeloma is available [11]–[15]. Glycosylation is primarily controlled by the concerted interaction of genes encoding glycosyltransferases and glycosidases. Analysis of the glycome at the transcriptional level should give insights on the potential expression of glycoconjugates. Knowledge about the genetic background of glycosylation processes which occur during tumor development may also provide valuable prospects for the design of new strategies for tumor drug development. Findings related to the glycosylation of syndecan 1 (CD138) underline this relevance: syndecan-1 consists of a core protein and covalently attached heparan-sulfate and chondroitin-sulfate chains which consist of linear N-acetylated glucosamine or alternating N-acetylated glucosamine and D-glucuronic acid units. Syndecan-1 acts as a “sponge” for heparin-sulfate binding growth, survival and communicational factors e.g. a proliferation-inducing-ligand (APRIL), epidermal growth factor (EGF) family members, insulin-like growth factor IGF-insulin-like growth factor binding proteins (IGFBP) or hepatocyte growth factor (HGF) [16]–[20]. It likewise attaches factors that are simultaneously involved in bone turnover, e.g. bone morphogenic protein-6 (BMP-6), or angiogenesis, e.g. vascular endothelial growth factor-A (VEGF-A), both produced by normal and malignant plasma cells [8], [9]. “Squeezing” this sponge by shedding of the heparan-sulfates from the syndecan-1 core protein, e.g. by heparanase produced by myeloma cells or the bone marrow environment [21], [22], can liberate these growth factors and promotes angiogenesis. The biological and clinical importance of genes involved in heparan- and chondroitin-sulfate synthesis is emphasized by our findings of EXT-1 heparan sulfate copolymerase (EXT1) being an adverse prognostic factor (14), and is critical for *in vitro* and *in vivo* growth of multiple myeloma [23].

Motivated by these observations, we present here a comprehensive analysis of the transcriptional glycome-expression in normal and malignant plasma cells. We relate this information to molecular entities in multiple myeloma regarding chromosomal aberrations and gene expression defined entities [24]–[26], clinical parameters, and survival.

## Patients, Materials and Methods

### Ethics statement

Patients presenting with previously untreated multiple myeloma (n = 332) at the University Hospitals of Heidelberg and Montpellier, and 10 healthy normal donors have been included in the study approved by the ethics committee (#229/2003 and S-152/2010) after written informed consent.

### Patients and healthy donors

Patients were diagnosed, staged, and response to treatment was assessed according to standard criteria [27]–[29]. Two hundred and forty seven patients underwent frontline high dose chemotherapy with 200 mg/m^2^ melphalan and autologous stem-cell transplantation. Survival data were validated by an independent cohort of 345 patients treated within the total therapy 2 (TT2) protocol [30]. Clinical parameters for the patients are provided in Table S1.

### Samples

Normal bone marrow plasma cells and myeloma cells were purified as previously published [9], [31]. The XG lines were generated at INSERM-UM1 U1040 as published [32]–[34], and the human myeloma cell lines U266, RPMI-8226, LP-1, OPM-2, SK-MM-2, AMO-1, JJN-3, NCI-H929, KMS-12-BM, KMS-11, KMS-12-PE, KMS-18, MM1.S, JIM3, KARPAS-620, L363 and ANBL6 were purchased from the German Collection of Microorganisms and Cell Cultures (Braunschweig, Germany) and the American Type Cell Culture (Manassas, VA), respectively, and cultured as recommended.

### Gene expression analysis

Gene expression profiling was performed as published [8], [9], [26], [35]. In brief, labeled cRNA was hybridized to U133 2.0 plus arrays according to the manufacturer's instructions (Affymetrix, Santa Clara, CA, USA). Expression data for myeloma cell samples are deposited in ArrayExpress under the accession numbers E-MTAB-317, E-GEOD-2658 and Gene Expression Omnibus GSE4581 (the latter two for the total therapy 2 (TT2) data and molecular class association, respectively). Quality controls were performed as previously published [26].

For qRT-PCR, cDNA synthesis was performed from 1 µg of total RNA after Oligo(dT) primed and first strand cDNA-synthesis was performed according to the manufacturer's guidelines (Super Script™ First –Strand Synthesis System for RT-PCR, Invitrogen, Karlsruhe, Germany). A total of 2.5 µl cDNA was amplified in 25 µl of SYBR Green PCR Master Mix (Applied Biosystems, Foster City, CA, USA) in the presence of 900 nmol of the specific primers for PIGM (fwd 5′: CCAGCCGGCGTCTTTGGTGT, rev 5′: CCCAGCAGCGGGGTGTAACG) using the 7300 Real Time PCR system (Applied Biosystems). Specifity of oligonucleotides (MWG Biotech, Ebersberg, Germany), was computer-tested (BLAST, NCBI) by homology search with the human genome. Samples were run in triplicate and experiments repeated twice. The thermal profile for the reaction was 2 min at 50°C, followed by 10 min at 95°C and then 40 cycles of 15 sec at 95°C and 1 min at 60°C. To exclude non-specific amplification, dissociation curve analysis was performed at the end of the run. Actin (primers fwd 5′: GCTCCTCCTGAGCGCAAG; rev 5′: CATCTGCTGGAAGGTGGACA) was chosen as endogenous reference. Relative gene expression was calculated using the comparative C_t_ method [36].

### Selection of target genes

The classification and nomenclature was used according to the Glycov4 chip of the consortium for functional glycomics (CFG, http://www.functionalglycomics.org) which contains 1259 genes and further adapted as described previously [37]. We focused in our analysis on a set of glycosylation relevant enzymes and proteins that belong to glycosyltransferases (GT), sulfo-transferases (ST), glycan-degrading enzymes (GD), genes necessary for sugar synthesis and transport (sugar s/t)) resulting in a gene list of 295 genes (Table S2A-C). Target genes were mapped to the Kyoto encyclopedia of genes and genomes (KEGG) database (http://www.genome.jp/kegg/pathway), Table S3. The 295 glycome-specific genes were found to be represented by 630 probe sets. Using the “presence absence calls with Negative Probesets” (PANP) algorithm, we identified 247 genes represented on 426 probe sets expressed at least in one sample [38]. We then selected 247 probe set/gene pairs choosing the probe set yielding the maximal variance and the highest signal. The flow of the data analysis is shown in Figure S1.

### Western blot

Proteins separated by SDS-PAGE were transferred to PVDF membranes (Millipore, Fl, USA) and subjected to Western blotting [39]. Western blots were incubated with primary antibodies rabbit anti-PIGM (Abgent, Aachen, Germany), rabbit anti-tubulin antibody (Biozol, Eching, Germany), and a mouse monoclonal antibody against protein disulfide isomerase (PDI), a marker for endoplasmatic reticulum, (Clone #34, Becton Dickinson (BD), Heidelberg, Germany). As secondary antibodies an anti-rabbit antibody (IgG) conjugated to horseradish peroxidase (POX) and an anti-mouse antibody (IgG+IgM) conjugated to POX (both antibodies from Dianova, Hamburg, Germany) were used. Stained protein bands were visualized by the ECL technique according to manufacturer's instructions and as performed previously [39].

### Interphase FISH

Interphase-FISH-analysis was performed on CD138-purified plasma cells as previously described [40] using probes for chromosomes 1q21, 9q34, 11q23, 11q13, 13q14.3, 15q22, 17p13, 19q13, 22q11, and translocations t(4;14)(p16.3;q32.3), t(11;14)(q13;q32.3). Presence of clonal/subclonal aberrations as well as the absolute number of chromosomal aberrations were defined as published [40]. The score of Wuilleme et al. [41] was used to assess ploidy. The percentage of malignant plasma cells was surrogated by the highest percentage of all tested chromosomal aberrations within this sample. For the analysis of molecular subentities, expression profiles of all patients presenting with the respective aberrations vs. all without were compared, independent of the presence of other aberrations.

### Flow cytometric analysis

Flow cytometric analysis of cell surface antigens was performed using a FACS Canto II and the FACSDIVA software (BD). Dead cells were excluded by staining with Via-Probe (BD). Cell surface staining was performed with an anti-human CD55 (clone 555693) and an anti-human CD59 (clone 555763) mouse monoclonal antibody, both conjugated to FITC or with an anti-human CD56 (clone 555518) mouse monoclonal antibody conjugated to APC (all from BD) for 30 min at 4°C. In order to remove the GPI-anchored CD55 and CD59 proteins from the cell surface KMS-12-BM and OPM-2 cells (1×10^6^ cells suspended in 400 µl PBS) were incubated with 40 µL phospholipase C (100 enzymatic units/mL; Life Technologies, Darmstadt, Germany) for 1 h at 37°C prior to antibody staining.

### Immunohistochemistry

Tissue sections were deparaffinized, subjected to heat induced epitope retrieval in TRS pH6,1 or pH9 (DAKO, Hamburg, Germany) and immunostained using a LSAB detection System (DAKO) as previously described [42].

### Statistical analysis

Expression data were gcrma-preprocessed [43] and log2 transformed. Unspecific filtering was applied selecting transcripts from the consensus list with at least a single present call [44] for further analysis. In case multiple probesets mapped to the same gene, the probeset showing highest standard deviation in the training cohort across samples was selected to represent the gene. Using this strategy, we selected 247 genes represented by 247 probe sets.

Unsupervised hierarchical clustering was performed using complete linkage and dissimilarity based on Euclidian distances. Cluster reliability was assessed using a bootstrap-based approach, which gives approximately unbiased probabilities. Differentially expressed genes between entities (normal bone marrow plasma cells, human myeloma cell lines, myeloma cells) as well as between myeloma samples with/without cytogenetic abnormalities were identified using the empirical Bayes approach based on the moderated t-statistic as implemented in the Bioconductor package limma [45]. Association between individual transcripts and overall survival/event-free survival was tested with Cox PH regression models. Prognostic gene expression and prognostic scores were dichotomized according to maximally selected log-rank statistic accounting for multiple testing due to cut-point search, and displayed using Kaplan-Meier estimates. Groups identified by this procedure were also used for subsequent multivariate analyses (see below). A classification rule to distinguish and predict samples of normal bone marrow plasma cells, human myeloma cell lines, and myeloma cells was determined with L1 penalized multinomial regression model, Again, using tenfold cross-validation to tune the hyperparameter lambda, and an additional leave-one-out loop to estimate misclassification in an unbiased fashion. Respective classifiers are available as supplementary files in Tables S4A and S5. For validation purposes, we used a) respective calculated thresholds, and b) the proportion determined in the test cohort.

All p-values were adjusted for multiple testing using Benjamini-Hochberg correction in order to control the false discovery rate. A prognostic gene signature for overall survival and event-free survival was developed using a L_1_ penalized Cox PH regression model with tenfold cross-validation for tuning the shrinkage parameter lambda. Internal validation was done with leave-one-out cross-validation based on an additional outer loop. External validation of prognostic transcripts and signatures was carried out on two publicly available data sets [46] (GSE24080) from the MAQC-II project. Multivariable Cox PH regression was used to adjust for clinic-pathological covariates. For illustration purpose, prognostic gene expression and prognostic scores were dichotomized according to maximally selected log-rank statistic accounting for multiple testing due to cut-point search, and displayed using Kaplan-Meier estimates. A classification rule to distinguish and predict samples of normal bone marrow plasma cells, human myeloma cell lines, and myeloma cells was determined with L_1_ penalized multinomial regression model, again using tenfold cross-validation to tune the hyperparameter lambda, and an additional leave-one-out loop to estimate misclassification in an unbiased fashion.

Differentially expressed genes were selected based on separate linear regression models for each gene (LIMMA), with MM/BMPC entity as explanatory variable and expression values as response, with the objective to identify any strongly regulated gene. Goeman's globaltest and L1 penalized regression models, MM/BMPC entity was used as response and gene expression levels as explanatory variables in a logistic regression model frame work. The reason to test entire (KEGG) gene sets rather than single genes with the global test in addition, is the possibility to detect the regulation of a biologically defined gene set, which might not necessarily be triggered by single large effect but perhaps cumulative moderate effects. The latter would not be detected with ordinary gene screening. So both approaches have slightly different objectives [47]. Corresponding p-values were adjusted for multiple testing using Bonferroni-Holm correction. All statistical tests were two-sided, p-values below 0.05 were considered statistically significant. All analyses were performed on the consensus gene list and carried out with R Version 2.14.0/Bioconductor Version 2.9 (http://www.R-project.org).

## Results

### Glycome expression in normal and malignant plasma cells

**Figure 1:**
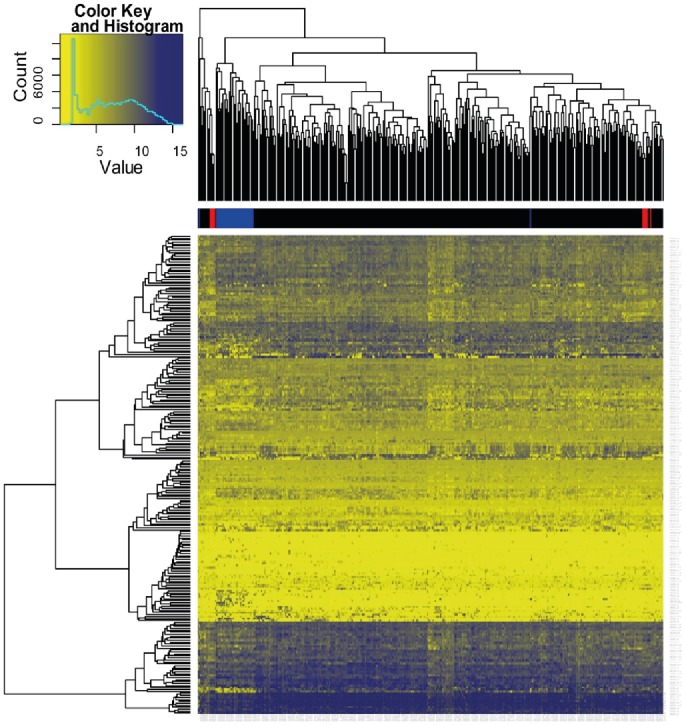
Unsupervised clustering of expression of glycan genes. Heat Map and global gene expression analysis: Unsupervised hierarchical clustering of glycan gene expression. Individual patient samples are shown in columns and genes in rows. Expression is displayed in yellow color and blue color depending on expression above or below median expression level. Color intensity is dependent on the degree of deviation from median. Sample type is highlighted above the heatmap in colors: black: myeloma cells, blue: human myeloma cell lines, red: normal bone marrow plasma cells.

First, we investigated whether normal and malignant plasma cells show a distinguishable glycome expression based on the 247 expressed glycome genes from our consensus list (Figure 1 and Table S2). Human myeloma cell lines (highlighted in blue color) all fell in a distinct cluster separated from normal bone marrow plasma cells (red color) and myeloma cells. Normal bone marrow plasma cells clustered tightly together in two clusters also containing myeloma cell samples. The stability of the normal bone marrow plasma cell cluster and the human myeloma cell line clusters were verified by bootstrapping (see methods), evidencing distinct entities. There were, however, no identifiable clusters within myeloma cell samples.

Next, we looked for significant differences between normal plasma cells, primary myeloma cells and human myeloma cell lines in the expression of 243 glycan-biosynthesis pathway genes as analyzed by empirical Bayes statistic and Benjamini-Hochberg correction for multiple testing (Table 1, Table S2). Of these, 60 showed a significantly higher, 20 a lower expression in myeloma compared to normal plasma-cell samples (*P*≤.05). Only 7 of the significantly upregulated genes are related to glycan degradation vs. 55 to glycome synthesis, indicating an increase in the pro-glycosylation part of the glycome.

**Table 1:** Upregulated genes and KEGG (Kyoto Encyclopedia of Genes and Genomes) pathways.

KEGG-pathway	Total genes tested	Number of significantly upregulated genes	Number of significantly downregulated genes	Percent upregulated of total	*P*-value of Goeman's global test of regulation MM vs BMPC
**GPI-anchor biosynthesis**	**7**	**3**	**0**	**43**	**9×10^−14^**
N-glycan glycosylation	35	13	1	37	6×10^−7^
Mucin-type O glycosylation	17	4	2	30	2×10^−7^
Other O-glycosylation	21	1	3	4	8×10^−3^
Other glycan degradation	9	2	0	22	8×10^−3^
**GAG-HS**	**7**	**4**	**2**	**57**	**2×10^−8^**
GAG-CS	20	5	3	25	4×10^−8^
GAG-KS	13	1	2	7	9×10^−5^
GAG degradation	12	5	1	42	7×10^−5^
GSL ganglio series	13	4	0	31	3×10^−5^
GSL lacto/neolacto series	21	2	3	9	2×10^−8^
GSL globo series	11	2	0	18	2×10^−2^

Using the Goeman's global test method the degree of distinction between myeloma cell samples (MM) and normal bone marrow plasma cell (BMPC) samples was investigated. The significance of p-level indicates the degree of difference between then BMPC and the MM samples. Glycan pathways were ordered according to the general families of macromolecular glycan structures, i.e. GPI-anchored glycoproteins, N- and O-glycosylated glycoproteins, glycosphingolipids (GSL) and proteoglycans (heparan sulfate (GAG-HS) glycosaminoglycans (GAG); chondroitin sulfate (GAG-CS) and keratin sulfate (GAG-KS). Most significant changes in the MM glycome were found in the GPI- and in the HS synthesis and were highlighted.

**Figure 2:**
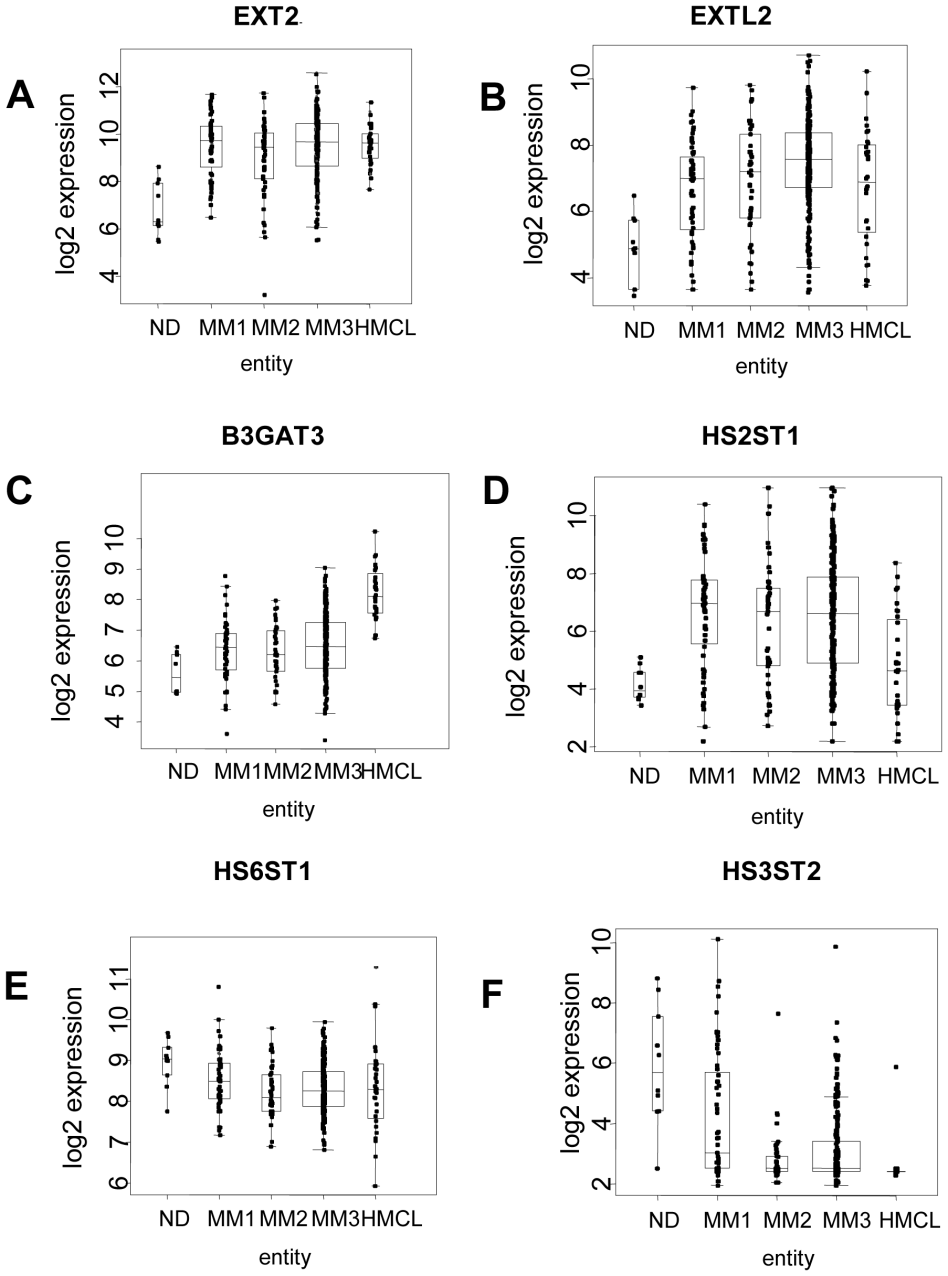
Gene expression of significantly regulated genes of the heparan sulfate pathway. **A–D**: Four genes of the glycosaminglycan-heparan sulfate (GAG-HS) biosynthetic pathway (*EXT2/EXTL2/B3GAT1/HS2ST1*) are significantly upregulated in myeloma compared to normal bone marrow plasma cells. Boxplots display the median and range for these genes for myeloma patients categorized into Durie-Salmon (DS) stage I-III (MM1-3), bone marrow plasma cells from normal donors (ND), and human myeloma cell lines (HMCL). **E,F**: Two heparan sulfotransferases (*HS6ST1* and *HS3ST1*) of the GAG-HS biosynthetic pathway are significantly downregulated in myeloma compared to normal plasma cells. Boxplots display the median and range for these genes for myeloma patients categorized into Durie-Salmon stage I-III (MM1-3), normal bone marrow plasma cells (ND), and human myeloma cell lines (HMCL).

**Figure 3:**
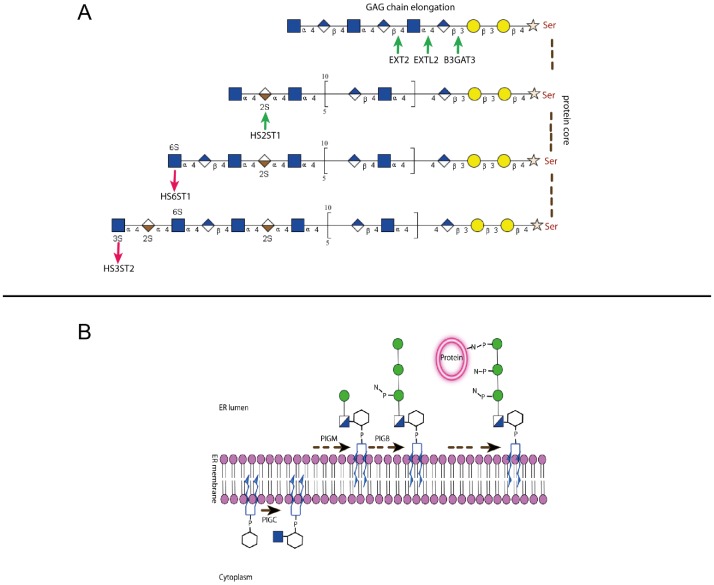
Steps of heparan sulfate and GPI anchor biosynthesis. **A**: Biosynthetical steps of the GAG-HS pathway and the functional role of the enzymes are shown. Green arrows indicate upregulated gene expression and red arrows downregulated gene expression. **B**: The steps of the GPI anchor biosynthesis are shown and the function of the proteins corresponding to the overexpressed *PIG*-genes M, C, B are shown.

The same holds true if gene families and subfamilies according to the KEGG database are analyzed by Goeman's global test between malignant and normal plasma cells as well as myeloma cell lines (Table 1, Table S3). Ranked by the degree of significance in differential expression, the glycosylphosphatidyl-inositol (GPI) -anchor pathway and the glycosaminoglycan heparan-sulfate (GAG-HS) pathway were identified as the top two pathways, in both cases showing a larger number of higher vs. lower expressed genes in GAG-HS pathway, 3 genes involved in the biosynthesis of the GAG basic structure and chain elongation were significantly higher expressed, two HS sulfotransferases responsible for sulfatation of the GAG-HS at various carbons of the GAG disaccharide unit were significantly lower and one higher expressed (Figure 2). An overview of the GAG-HS biosynthetical pathway is provided in Figure 3A highlighting the modulated genes.

**Figure 4:**
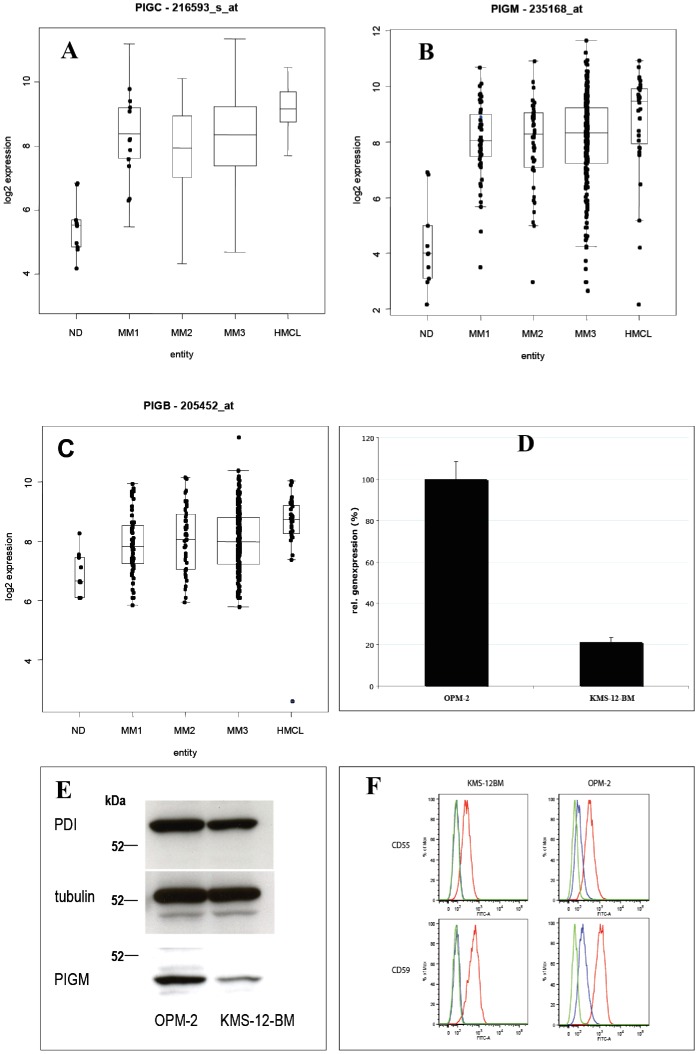
Gene expression of significantly overexpressed genes related to the glycosylphosphatidylinositol (GPI) anchor pathway. **A–C:** Median gene expression of phosphatidylinositol glycan anchor biosynthesis, class (*PIG*) -M, - C, B is significantly higher in malignant vs. normal bone plasma cells. Boxplots display the median and range for these genes for myeloma patients categorized into Durie-Salmon stage I-III, myeloma is compared to results in normal bone marrow plasma cells (ND), and human myeloma cell lines (HMCL). **D/E:** Validation of *PIG-M* gene expression using RT-PCR and Western Blotting using the HMCL OPM-2 with a high PIG-M expression and the HMCL KMS-12-BM with a low PIG-M expression as per gene expression profiling. **D:** mRNA expression of *PIG-M* gene was investigated using RT-PCR. **E:** Western Blotting was performed using standard markers tubulin and PDI, a marker for endoplasmatic reticulum, to confirm equal loading of gel and investigate the PIG-M protein of OPM-2/PIG-M expression high (left lane) and the KMS-12-BM/PIG-M expression low (right lane). **F:** Flow cytometric analysis of CD55 and CD56 on KMS-12-BM and OPM-2 myeloma cell lines with and without prior PLC treatment. Red lines: CD55/CD59 expression without PLC treatment, blue lines: CD55/CD59 expression after PLC-treatment, green lines: background staining solely with secondary antibody coupled to FITC.

**Figure 5:**
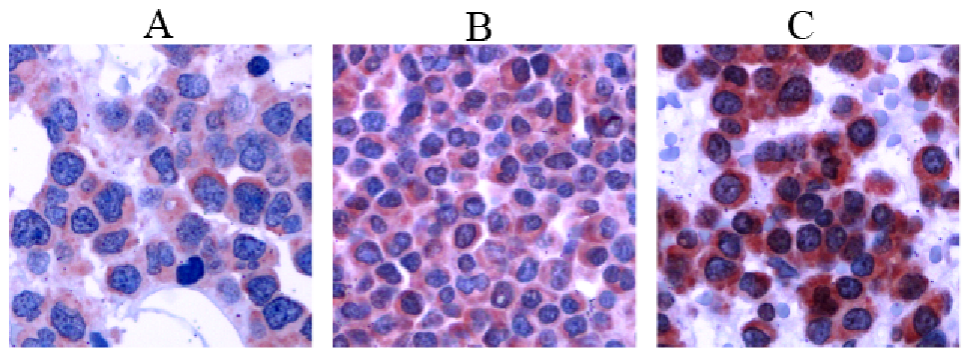
Expression of PIGM in myeloma cells. Immunohistochemical staining of PIGM on primary myeloma cells showing different staining patterns. A: weak, B: moderate, and C: strong.

Three genes involved in the first steps of the glycosylphosphatidylinositol (GPI)-anchor biosynthesis encoding the enzymes phosphatidylinositol glycan anchor biosynthesis, class M, B and C (*PIGM*, *PIGB* and *PIGC*) (see Figure 4D for the biosynthetical steps catalyzed by the encoded enzymes), were two to four-fold higher expressed in malignant vs. normal plasma cells (Figure 4). Of these genes, *PIGM* expression, encoding for the enzyme phosphatidylinositol glycan anchor biosynthesis protein M (PIG-M), was significantly higher in myeloma cell samples with 1q21-gain or del13q14 and significantly lower in hyperdiploid myeloma cell-samples. Transcriptional data for *PIGM* expression were validated by qRT-PCR and for the corresponding PIG-M protein expression by Western blotting using the myeloma cell lines KMS-12-BM and OPM-2 with low and high *PIGM* gene expression, respectively (Figure 4D, E). To investigate whether diminished PIG-M expression impacts cell surface expressed GPI-anchored receptors, we analyzed KMS-12-BM and OPM-2 for the expression of complement regulatory proteins CD55 (Decay Accelerating Factor) and CD59 [48], [49]. Dependence of their cell surface expression by GPI-anchoring was verified by phospholipase C (PLC) treatment prior to antibody staining. On both cell lines CD55 and CD59 were susceptible to PLC treatment as their expression was significantly diminished after enzymatic cleavage (Fig. 4F). As expected, CD55 and CD59 expression was considerably lower on PIG-M^low^ KMS-12-BM cells as compared to PIG-M^hi^ OPM-2 (CD55: mean fluorescence intensities 278 and 624, respectively; CD59: mean fluorescence intensities 398 and 1185, respectively). CD56 (NCAM), although described as being occasionally expressed as GPI-anchored variant, was only present as the membrane-spanning type on these myeloma cell lines (data not shown). Thus, varying expression of PIG-M on myeloma cells has a direct influence on cell surface expression of functionally relevant surface antigens. PIGM expression was also validated in a set of 21 primary MM samples by immunohistochemistry, where we could differentiate a i) strong, ii) moderate, and iii) weak staining pattern (Figure 5).

An overview of the phosphatidylinositol glycan anchor biosynthesis pathway is provided in Figure 3B highlighting the modulated genes.

### Glycome-gene expression specific classifier for multiple myeloma

The difference in the expression pattern of the different populations was further emphasized by the ability of a Lasso-classifier based on 19-glycome genes which allows predicting a sample as myeloma cell vs. normal plasma-cell sample with a specificity of 0.70 and a sensitivity of 1.0 (Table S4A and B).

### Association of glycome-gene expression with genetically defined subentities of multiple myeloma

**Figure 6:**
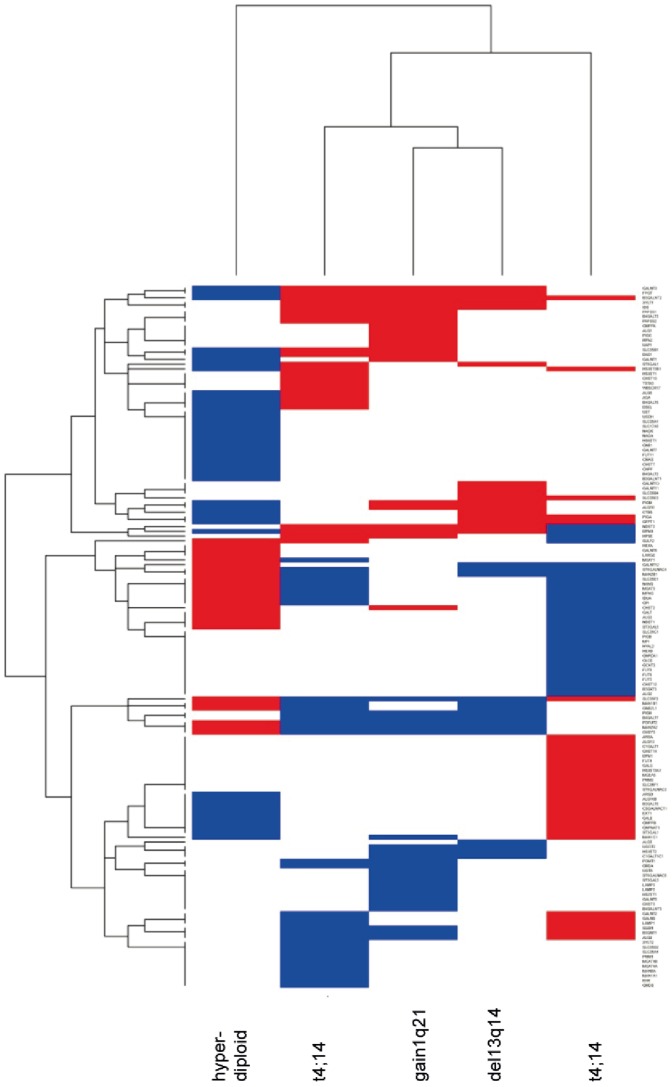
Heat Map for genes significantly regulated comparing cytogenetically defined myeloma subentities. A subset of glycan genes was identified by investigating significance of gene regulation for patients with the respective cytogenetic aberration compared to patients without the aberration. In a next step all identified genes (n = 148) were clustered (genes shown in rows) with the corresponding cytogenetic aberration displayed in vertical orientation (hyperdiploid (HRD), t(4;14), t(11;14), del13q14, gain of 1q21). Blue color indicates significantly downregulated genes, red color indicates significantly upregulated genes.

We tested for associations of the glycome-specific genes with chromosomal aberrations as assessed by iFISH. For the analysis of molecular subentities, expression profiles of all patients presenting with the respective aberrations were compared to those without independent of the presence of other aberrations. We found significant differences in the median gene expression level between patients with either t(4;14), t(11;14), hyperdiploid myeloma, or presence of either a deletion 13q14, or gain of 1q21 vs. patients' myeloma cell-samples not carrying the respective abnormality. Interestingly, the groups with prognostically adverse aberrations t(4;14), 1q21-gain and del3q14 displayed substantial similarities in the expression pattern, as did the groups with risk neutral or rather favorable cytogenetics, *i.e.* t(11;14) and hyperdiploid myeloma (Figure 6, hyperdiploid group (HRD)).

**Figure 7:**
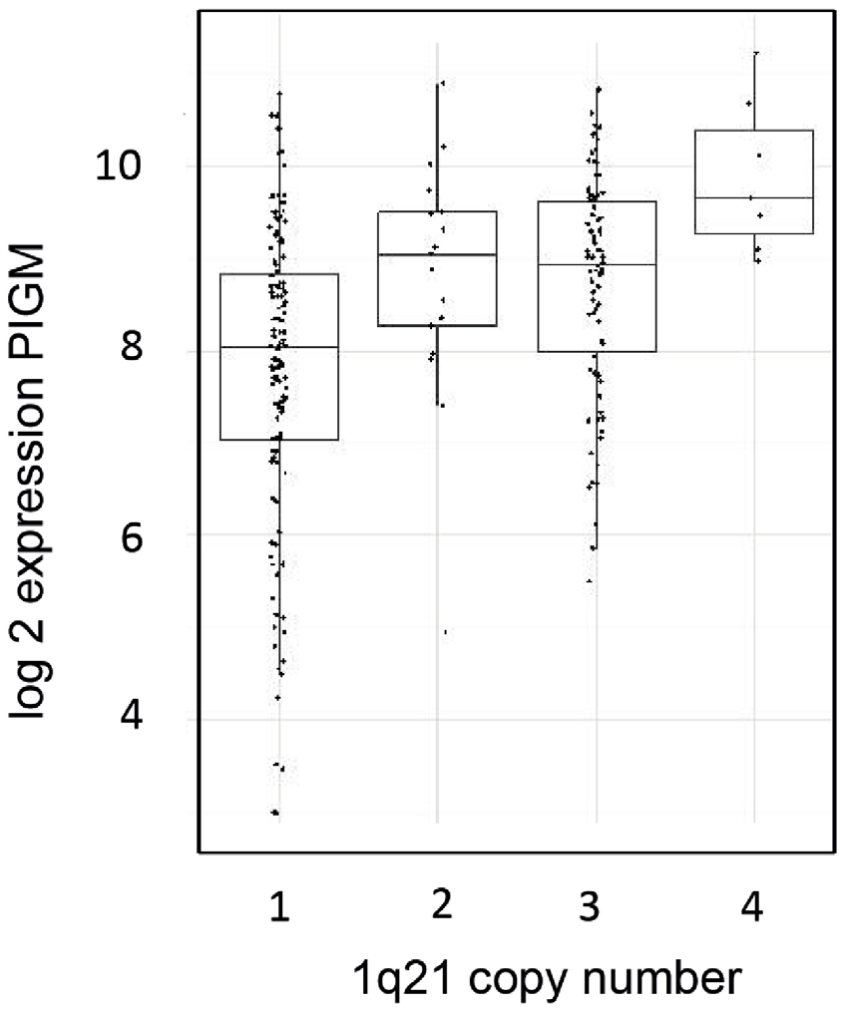
*PIGM* expression correlated to 1q21 copy number. *PIGM* gene expression was plotted as log 10 and correlated with cytogenetically determined 1q21 gene copy number.

*PIGM*, one of the most highly overexpressed genes in the comparison between normal and malignant plasma cells, and contributing to the classifier distinguishing between both, was also one of the overexpressed genes in the myeloma group with the adverse cytogenetic markers. We therefore further investigated the association between *PIGM* and 1q21 and found a constant increase in *PIGM* expression related to the copy number of 1q21 (Figure 7).

### Glycome gene expression and survival

**Figure 8:**
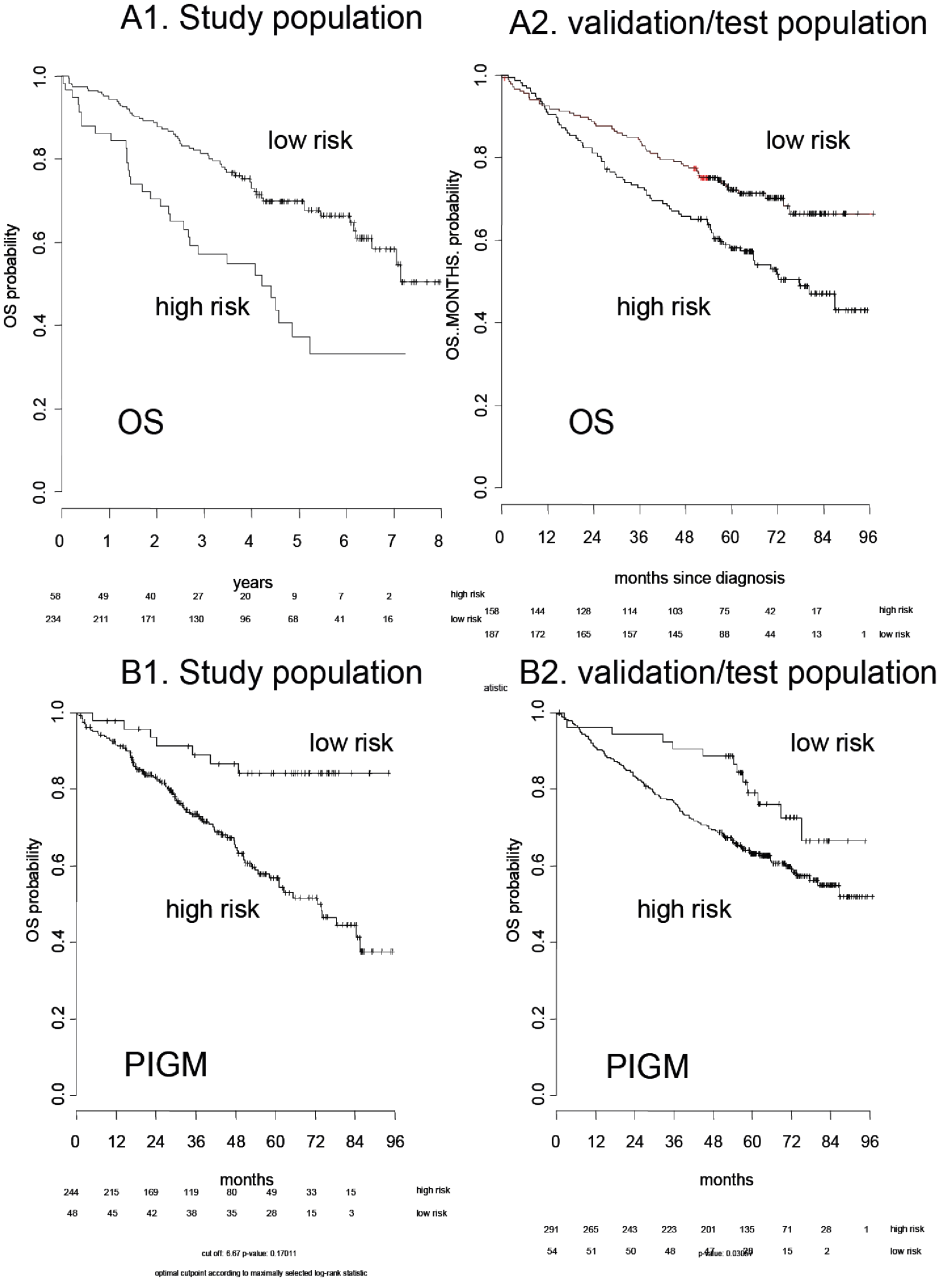
Prognostic value of glycan-gene expression score for survival. **A**. **Gene Score:** using a LASSO classifier model a 38-gene-score (Table S5) was identified that had significant prognostic value for overall survival for our population of 332 multiple myeloma patients (A1, *P* = 0.002, HR1.84) and the TT2-test population (A2, *P*<0.0001, HR 1.58). Overall survival curves are shown according to Kaplan-Meier. **B. Prognostic value of phosphatidylinositol glycan anchor biosynthesis, class M (PIG-M) gene expression for survival:** Using a CoxPH model expression to investigate for glycan gene expression with significance for OS the phosphatidylinositol glycan anchor biosynthesis, class M (*PIG-M*) gene was found to be statistically significant for overall survival as shown for our patient population (B1, *P*<0.001, HR 1.28) and the test population (B2, *P* = 0.004, HR 1.19).

We sought to determine if glycome expression profile could be prognostic for event-free and overall survival. For this reason we used a penalized Cox proportional hazard regression model to identify a set of glycome gene associated with survival. With this approach, we identified a 38-glycan gene signature significantly associated with overall survival (*P*<.01, HR 1.45; Table S5 displaying the gene signature). *PIGM* as part of the GPI-anchor biosynthesis pathway was the most prominent gene higher expressed in the adverse prognosis group. For details, see Table S5. Next we investigated the prognostic relevance of the glycan gene signature for survival using the TT2 cohort and found a significant prognostic overall survival impact in this validation cohort (*P*≤.001, HR 1.51) (Figure 8A). The prognostic significance of the glycome gene signature is independent of the presence of a t(4;14) as predicted by gene expression profiling or the ISS-stage in a multivariate analysis in the (validation) TT2-cohort (*P* = 0.02). Interestingly, a glycome-gene score for event-free survival could not be delineated.

To determine the prognostic significance of the expression of individual glycome-associated genes, we used a univariate Cox proportional hazard model with Benjamini-Hochberg correction for multiple testing. The gene coding for the GPI-anchor biosynthesis enzyme PIG-M was the only gene singly associated with overall survival in our population (*P*≤.001, HR 1.31). The prognostic impact of *PIGM* expression was also detected in a multivariate analysis including the international staging system and t(4;14) (*P*<.01). The validation of this result was performed on the TT2 cohort and *PIGM* expression was confirmed to be a significant prognostic parameter in univariate (*P* = 0.004, HR 1.19; Figure 8 B2) tests. No gene was significantly associated with event-free survival.

## Discussion

Two major pathways identified in our analysis, i.e. the biosynthesis of GPI-anchored glycoproteins and the synthesis of glycosaminoglycans as the sugar moiety of proteoglycans, are of particular interest in myeloma. Glycome genes involved in GPI anchor biosynthesis have not been studied in detail in normal or malignant plasma cells. Several encoded proteins potentially associated with myeloma pathogenesis require GPI anchors [50]. The functional role of these membrane proteins in myeloma biology ranges from cell adhesion, immune escape (complement inhibition, CD59) to modification of cell signaling (glypicans) [51].

Interestingly, despite being significantly predictive for overall survival on two independent cohorts of patients, neither the glycan gene score nor *PIGM* as single gene reached statistical significance for event-free survival. This is in agreement with glycan-dependent aspects of myeloma pathophysiology not being a main (at least not direct) target of current first-line therapies. The prognostic relevance for OS might then rather be driven by addition of rather small incremental effects in each line of treatment. This further fosters the potential therapeutic interest in strategies based on interfering with glycosylation events or pathways involving glycosylation.

PIGM expression as validated on protein level using Western blotting, flow cytometry and immunohistochemistry is associated with CD55/CD59 expression on myeloma cell lines and thus impacts on cell surface expressed GPI-anchored receptors. The concerted upregulation of specific GPI-anchored proteins and enzymes responsible for the synthesis of the GPI anchor itself in myeloma cells clearly deserves more attention in future studies.

The second most prominent regulated pathways on gene expression level are those of GAG synthesis, in particular those for heparan sulfate and chondroitin sulfate synthesis. GAG consist of repetitive disaccharide units which are modified by sulfatation or acetylation in O- or N- linkage at various carbons along the GAG chain. GAG either as heparan sulfate or chondroitin sulfate chains or in combination are covalently attached to protein cores of the respective proteoglycans. Due to the large heterogeneity within the carbohydrate moiety and the different sizes and numbers of GAG chains proteoglycans represent a very diverse group of macromolecules implying a multitude of functions in cell adhesion, signaling and regulation of soluble proteins. The proteoglycan Syndecan-1 (CD138), the hallmark of normal and malignant plasma cells, is covered with covalently bound heparan and chondroitin sulfate side chains [14] allowing it to act as most important “sponge” for heparan sulfate binding myeloma growth and survival factors such as VEGF or IGF-1, as well as factors impacting in bone turnover, e.g. osteoprotegerin (OPG), a decoy receptor for receptor activator of NFκB ligand thus preventing the OPG-mediated inhibition of osteoclastic bone resorption [52]. These factors can be liberated by heparanase, produced by the bone marrow microenvironment as well as some myeloma cell samples, which cleaves heparan sulfate chains from proteoheparan sulfates [22]. Heparan sulfate side chains play an important role in other proteoglycans like glypicans [51]. We recently described the “heparan sulfate code” impacting on ligand binding capacity of heparan sulfate proteoglycans and the relevance of sulfatation of the heparan sulfate chains, in particular by the sulfatases SULF1 and SULF2 which selectively remove 6-O-sulfate from heparan sulfate chains thereby altering binding sites for signaling molecules [14], [15]. We confirm these data in our current analysis, adding evidence for a lower expression of 3-O- and 6-O-sulfatation within the core region of the GAG chains by reduced activity of the corresponding sulfotransferases heparan sulfate O-6 sulfotransferase-1 (*HS6ST1*) and heparan sulfate O-3 sulfotransferase-2 (*HS3ST2*). Enzymes relevant for the initiation of GAG formation and heparan sulfate chain elongation, beta-1,3-glucuronyltransferase 3 (*B3GAT3*), multiple exostosin-like 2 (*EXTL2*) and *EXT2* heparan sulfate copolymerase (*EXT2*), display enhanced expression in myeloma cells. In particular, *EXT2* has been identified in earlier studies as a glycome gene differentially expressed in myeloma cells as compared to normal plasma cells which may have a crucial role in multiple myeloma growth development [14]. Thus, mainly core rather than terminal sulfatation of the heparan sulfate side chain are required for the ligand binding activity contributes to malignant plasma cell phenotype. The relevance of regulated enzymatic activity for proteoheparansulfate synthesis has been demonstrated by knockdown of *EXT1* also involved in the chain elongation of heparan sulfate chains [23], [53] leading to reduced growth and increased apoptosis in myeloma cells.

In addition to the GAG heparan sulfate the related GAG chondroitin sulfate pathway is upregulated in myeloma cells. We have shown that the serglycin family of proteochondroitinsulfates actually constitutes the main proteoglycans in human malignant B-cells. As serglycins support immune escape mechanisms by inhibition of complement mediated lysis and inhibit bone mineralization they could have role in multiple myeloma pathophysiology as well [54], [55]. Remarkably, chondroitin sulfate chains of myeloma proteoglycans contained mostly chondroitin-4-sulfate but also stretches of non-sulfated chondroitin sulfate which may point to distinct patterns of sulfation possibly relevant for specific binding of preferentially soluble proteins. Also, some GAG chains of myeloma cells consisted of hybrid heparan/chondroitinsulfates [56].

The analysis of gene expression profiles with special regard to the glycome gave us the opportunity to investigate more than 300 samples with long clinical follow-up dating back to the early 2000s. In particular, we could investigate whether the glycosylation process is relevant for the biology, prognostication and treatment of myeloma. A striking feature was the unexpected yet close correlation of GPI-enzymes, like *PIGM* with survival. In a next step the relevance of these transcriptional events has to be validated for the translational level of the enzymes and the glycosylation pattern of the respective end products. As an example, we have shown for *PIGM*, transcription of this enzyme is closely correlated with its translation in distinct multiple myeloma cell lines. The expression of GPI-anchored proteins with known functions in myeloma cells, such as CD55 and CD59 [48], [49] has been demonstrated. Based on our findings, we see the continuation of our work in a subsequent in-depth-analysis of specific targetable pathways and their role for myeloma treatment.

Taken together we demonstrate here for the first time the glycome expression of malignant plasma cells to be significantly different compared to their normal counterpart, on the level of single gene expression as well as gene-family-wise comparison. This signature is strong enough that samples can be predicted to be normal or malignant plasma cells on the basis of glycome expression alone. Additionally, glycome expression is associated to genetic subentities as identified by chromosomal aberrations, i.e. t(11;14), t(4;14), hyperdiploidy, 1q21-gain, and deletion of 13q14. The biological relevance of the glycome-expression is further emphasized by the impact regarding overall survival for the derived signature as well as expression of single genes, *i.e.* the GPI-anchor pathway *PIGM* gene, both being independent of adverse prognostic factors like ISS-stage or presence of t(4;14) in multivariate analysis. Furthermore, the most prominently different pathways between normal and malignant plasma cells are of high biological relevance and at the same time represent potential drugable target.

## Supporting Information

Figure S1 **Flow of data analysis.**
(TIF)

Table S1 **Patient characteristics (n = 331).**
(DOC)

Table S2 **A. List of significantly upregulated genes. B. List of significantly downregulated genes. C. List of genes not significantly different between multiple myeloma and normal donors (BMPC).** All genes included in this analysis are displayed with common name and subcategory and the mean expression level for myeloma patients and normal donors. Genes are presented in blocks depending on the significance of up and down regulation comparing MM and bone marrow plasma cells (BMPC).
(DOC)

Table S3 **Overview of glycome genes categorized by gene family and subfamily.**
(DOC)

Table S4 **A. Genes involved in Lasso classifier to distinguish multiple myeloma vs. bone marrow plasma cells (BMPC).** Genes relevant for the classification of MM vs BMPC samples are shown in the right column. The relative weighting of each gene for the classifier is shown on the right column. The intercept for this classifier is 6.29. **B. Sensitivity/Specificity analysis of Lasso classifier.** Results of the classification of samples resulting in best possible sensitivity and high specificity for classification.
(DOC)

Table S5 **38-gene signature correlating with overall survival.** Using a penalized Cox proportional hazard regression model we identified a 38-glycan gene signature significantly associated with overall survival (OS; *P*<0.001, Figure 4). The absolute value of the LASSO coefficient indicates the weight of the respective gene for the gene score. For genes in which upregulation contributed to high risk condition, the *PIG-M* gene of the GPI-anchor biosynthesis pathway and the *CHSY1* gene of the GAG chondroitin sulfate pathway had the highest coefficients. Negative or positive values of the LASSO coefficient indicate if down or upregulation of the respective gene contributed to the high risk profile. For genes in which downregulation contributed most to high risk profile 2 glycan degradation genes were identified.
(DOC)
