# Supplementary figures and images for: The Glycome of Normal and Malignant Plasma Cells
Source: PLoS One. 2013 Dec 26;8(12):e83719. doi: 10.1371/journal.pone.0083719 (PMC3873332; doi:10.1371/journal.pone.0083719)

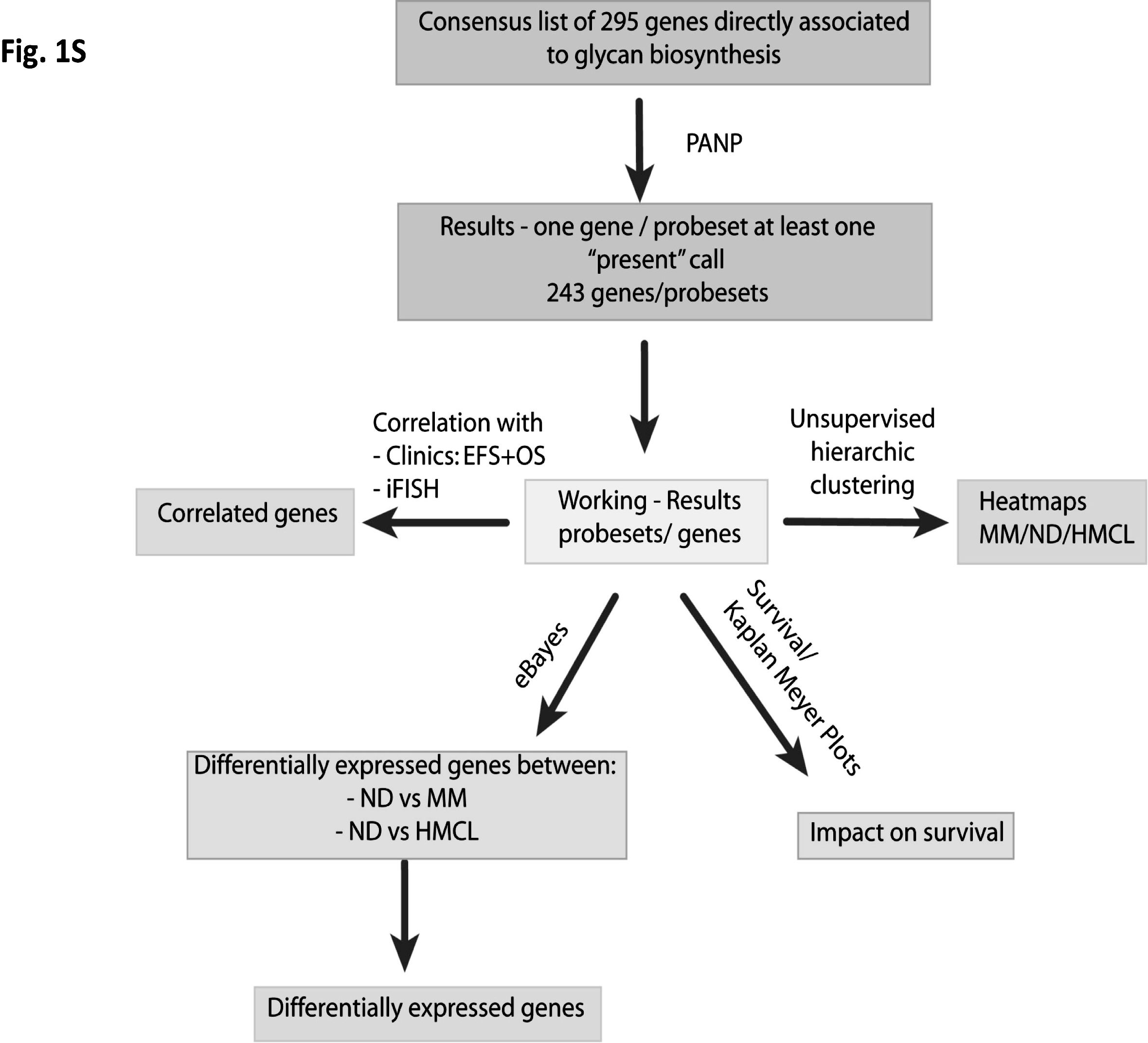

Supplement: Figure S1 — Flow of data analysis. (TIF) [file pone.0083719.s001.tif]
