# Supplementary material for: The Glycome of Normal and Malignant Plasma Cells
Source: PLoS One. 2013 Dec 26;8(12):e83719. doi: 10.1371/journal.pone.0083719 (PMC3873332; doi:10.1371/journal.pone.0083719)
Supplement: Table S1 — Patient characteristics (n = 331). (DOC) [file pone.0083719.s002.doc]

**Supplemental Table S1: Patient characteristics (n=331)**

Char acteristic Parameter

Female [n, %] 144 (43.5)

age [mean in years] 58

ISS stage (n, %)

1. 159 (49.0)
2. 102 (31.7)
3. 62 (18.9)

β_2_-microglobulin >3.5 μg/ml (n) 122

Multiple Myeloma [n] 331

stage DS I 59

stage DS II 46

stage DS III 225

Monoclonal Protein [n]

IgG 194 (58.6)

IgA 79 (23.8)

Bence-Jones 48 (14.5)

Ascretory 6 (1.8)

IgD 4 (1.2)

t4;14 [n] 47 (18.2)

gain 1q21 [n] 103 (42.6)

del 13q14 [n] 137 (52.3)

del 17p [n] 34 (13.4)
