# Supplementary material for: The Glycome of Normal and Malignant Plasma Cells
Source: PLoS One. 2013 Dec 26;8(12):e83719. doi: 10.1371/journal.pone.0083719 (PMC3873332; doi:10.1371/journal.pone.0083719)
Supplement: Table S2 — A. List of significantly upregulated genes. B. List of significantly downregulated genes. C. List of genes not significantly different between multiple myeloma and normal donors (BMPC). All genes included in this analysis are displayed with common name and subcategory and the mean expression level for myeloma patients and normal donors. Genes are presented in blocks depending on the significance of up and down regulation comparing MM and bone marrow plasma cells (BMPC). (DOC) [file pone.0083719.s003.doc]

**Supplemental Table S2A: list of significantly upregulated genes**

| **Common.name** | **Sub.category** | **mean_log2.**  **MM** | **mean_log2.**  **BMPC** |
| --- | --- | --- | --- |
| ARSB (Arylsulfatase B precursor ) | Arylsufatases | 7,8 | 6 |
| GALNT1 (ppGalNAc T1; polypeptide N-acetylgalactosaminyltransferase 1) | GalNAc-T | 10,3 | 8,6 |
| GALNT3 (polypeptide N-acetylgalactosaminyltransferase 3) | GalNAc-T | 9,1 | 6,8 |
| GALNT7 ( polypeptide N-acetylgalactosaminyltransferase 7) | GalNAc-T | 5,3 | 3,2 |
| B3GALNT2 (beta-1 3-N-acetylgalactosaminyltransferase 2) | Gal-T | 10,1 | 9,5 |
| B4GALT3 (UDP-Gal:betaGlcNAc beta 1,4- galactosyltransferase, polypeptide 3) | Gal-T | 11,9 | 11,2 |
| C1GALT1 (core 1 synthase ) | Gal-T | 7,5 | 5,8 |
| CHSY1 (carbohydrate (chondroitin) synthase 1) | Gal-T | 12,1 | 11,3 |
| CHSY-2 (chondroitin sulfate synthase 3) | Gal-T | 8,3 | 4,5 |
| UGT8 [UDP glycosyltransferase 8 (UDP-galactose ceramide galactosyltransferase)] | Gal-T | 6,5 | 2,7 |
| DPAGT1 [dolichyl-phosphate (UDP-N-acetylglucosamine) N-acetylglucosaminephosphotransferase 1 (GlcNAc-1-P transferase)] | GlcNAC-T | 10,6 | 9,9 |
| EXTL2 (multiple exostoses-like 2) | GlcNAc-T | 7,2 | 4,8 |
| MGAT1 (mannosyl (alpha-1,3-)-glycoprotein beta-1,2-N-acetylglucosaminyltransferase) | GlcNAc-T | 11,8 | 11,1 |
| PIGC [phosphatidylinositol glycan anchor biosynthesis, class C] | GlcNAc-T | 8,1 | 5,5 |
| ALG10 [asparagine-linked glycosylation 10 homolog (yeast, alpha-1,2-glucosyltransferase)] | Glc-T | 6,9 | 5,2 |
| ALG6 [asparagine-linked glycosylation 6 homolog (S. cerevisiae, alpha-1,3-glucosyltransferase)] | Glc-T | 9,6 | 8 |
| ALG8 [asparagine-linked glycosylation 8 homolog (S. cerevisiae, alpha-1,3-glucosyltransferase)] | Glc-T | 11,8 | 9,9 |
| UGCG [ceramide glucosyltransferase] | Glc-T | 6,7 | 4,1 |
| B3GAT3 [beta-1,3-glucuronyltransferase 3 (glucuronosyltransferase I)] | GlcUA-T | 6,4 | 5,5 |
| HPSE (Heparanase) | Heparanases | 5,1 | 3,1 |
| HEXB (hexosaminidase B preproprotein) | Hexosamini  Dase | 13,2 | 12,5 |
| HEXA [hexosaminidase A (alpha polypeptide)] | Hexosaminidase | 10,5 | 9,4 |
| EXT2 HS Copolymerase [GAG Enzyme] | HS GlcNAc/GlcA Transferase | 9,4 | 6,8 |
| HYAL2 (hyaluronoglucosaminidase 2) | Hyalurono-glucosaminidases | 4,9 | 3,4 |
| ASAH1 [N-acylsphingosine amidohydrolase (acid)] | Lysozomal  Enzymes/  Proteins | 10 | 8,4 |
| LAMP2 (lysosomal-associated membrane protein 2) | Lysozomal  Enzymes/  Proteins | 6,6 | 5,3 |
| LAMP3 (lysosomal-associated membrane protein 3) | Lysozomal  Enzymes/  Proteins | 11,5 | 8,9 |
| MAN1B1 [Mannosidase, alpha, class 1B, member 1] | Mannosidase | 8 | 6 |
| MAN2A1 (mannosidase, alpha, class 2A, member 1) | Mannosidase | 11,4 | 8,5 |
| ALG09 | Man-T | 8,7 | 7,5 |
| ALG1 | Man-T | 8,7 | 7,5 |
| ALG3 | Man-T | 10,9 | 9,6 |
| PIG B | Man-T | 8 | 6,9 |
| PIGM phosphatidylinositol glycan anchor biosynthesis | Man-T | 8,1 | 4,3 |
| SLC17A5 [solute carrier 17 (anion/sugar)] | Miscellaneous | 6,6 | 5,2 |
| DAD 1 | N-glycans-  transferase | 12,6 | 11,9 |
| RPN1 (ribophorin I) | N-glycans-  transferase | 13 | 11,5 |
| SLC35A1 [solute carrier family 35 (CMP-sialic acid transporter), member A1] | Nuc. Sugars  Transporters | 11 | 9,9 |
| SLC35A3 [solute carrier family 35 (UDP-N-acetylglucosamine (UDP-GlcNAc) transporter), member A3] | Nuc. Sugars  Transporters | 5,4 | 4,3 |
| SLC35A4 [solute carrier family 35, member A4] | Nuc. Sugars  Transporters | 9,2 | 8,3 |
| SLC35A5 [solute carrier family 35 member A5] | Nuc. Sugars  Transporters | 9,8 | 8,8 |
| SLC35B2 [solute carrier family 35 member B2] | Nuc. Sugars  Transporters | 9,1 | 7,3 |
| SLC35B4 [solute carrier family 35 member B4] | Nuc. Sugars  Transporters | 8,5 | 5,2 |
| SLC35D1 [solute carrier family 35 (UDP-glucuronic acid/UDP-N-acetylgalactosamine dual transporter), member D1] | Nuc. Sugars  Transporters | 8,4 | 5,4 |
| SLC35E3 [solute carrier family 35 member E2] | Nuc. Sugars  Transporters | 9,1 | 8,2 |
| CMAS [cytidine monophosphate N-acetylneuraminic acid synthetase] | Nucleotide  Synthesis | 7,5 | 6,3 |
| GALK2 [galactokinase 2] | Nucleotide  Synthesis | 7,8 | 5,4 |
| GNB1 [guanine nucleotide binding protein beta-1] | Nucleotide  Synthesis | 11,4 | 9,9 |
| GPI [glucose phosphate isomerase] | Nucleotide  Synthesis | 10,9 | 8,9 |
| KHK [ketohexokinase] | Nucleotide  Synthesis | 7 | 6,1 |
| PAPSS1 [3'-phosphoadenosine 5'-phosphosulfate synthase 1] | Nucleotide  Synthesis | 11,7 | 10,3 |
| PGM1 [phosphoglucomutase 1] | Nucleotide  Synthesis | 10,8 | 9,5 |
| TSTA3 [tissue specific transplantation antigen P35B] | Nucleotide  Synthesis | 9,1 | 8 |
| UGDH [UDP-glucose dehydrogenase] | Nucleotide  Synthesis | 10,3 | 9,5 |
| ST3Gal5 [ST3 beta-galactoside alpha-2,3-sialyltransferase 5] | Sia-T | 11 | 9,2 |
| ST3Gal6 [ST3 beta-galactoside alpha-2,3-sialyltransferase 6] | Sia-T | 11,7 | 8,3 |
| ST6GalNAc6 [ST6 (alpha-N-acetyl-neuraminyl-2,3-beta-galactosyl-1,3)-N-acetylgalactosaminide alpha-2,6-sialyltransferase 6] | Sia-T | 5,4 | 5,1 |
| ST8SIA4 [ST8 alpha-N-acetyl-neuraminide alpha-2,8-sialyltransferase 4] | Sia-T | 9,8 | 7,2 |
| CHST11 [carbohydrate (chondroitin 4) sulfotransferase 11] | Sulfo-T | 8,7 | 6,2 |
| CHST14 [carbohydrate (N-acetylgalactosamine 4-0) sulfotransferase 14; aka dermatan 4 sulfotransferase 1] | Sulfo-T | 6 | 5,2 |
| HS2ST1 [heparan sulfate 2-O-sulfotransferase 1] | Sulfo-T | 6,5 | 4,1 |

**Supplemental Table S2B: list of significantly downregulated genes**

| **Common.name** | **Sub.category** | **MM mean_ log2.** | **mean_ log2.**  **ND** |
| --- | --- | --- | --- |
| FUT4 ( fucosyltransferase 4) | Fucosyl-T | 3,2 | 4,5 |
| ChGn (CSGalNAcT1/ChGalNAcT1; chondroitin beta1 4) | GalNAc-T | 10,7* | 12,7 |
| GALNACT-2 (CSGalNAcT2/ChGalNAcT2;chondroitin beta1 4) | GalNAc-T | 10,3 | 12,1 |
| GALNT2 (polypeptide N-acetylgalactosaminyltransferase 2) | GalNAc-T | 6,7 | 8,5 |
| B4GALNT4 (beta-1 4-N-acetyl-galactosaminyl transferase 4) | GalNAc-T | 3,8 | 4,8 |
| B4GALT1 (UDP-Gal:betaGlcNAc beta 1,4- galactosyltransferase, polypeptide 1) | Gal-T | 10 | 12,1 |
| ABO (transferase A, alpha 1-3-N-acetylgalactosaminyltransferase; transferase B, alpha 1-3-galactosyltransferase). | Gal-T/GalNAc-T | 5,6* | 6,2 |
| OGT [O-linked N-acetylglucosamine (GlcNAc) transferase | GlcNAc-T | 6,6 | 10,4 |
| NPL (N-acetylneuraminate pyruvate lyase) | Mannosidases | 2,7 | 4,4 |
| PPBP (pro-platelet basic protein) | Miscellaneous | 5,7* | 10 |
| PMM2 [phosphomannomutase 2] | Nucleotide Synthesis | 9,4 | 10,1 |
| GMPPA [GDP-mannose pyrophosphorylase A] | Nucleotide Synthesis | 7,1 | 8,3 |
| GALK1 [galactokinase 1] | Nucleotide Synthesis | 2,5 | 4,8 |
| GALNS (N-acetylgalactosamine-6-sulfatase precursor) | Sulfatases | 6* | 6,8 |
| HS6ST1 [heparan sulfate 6-O-sulfotransferase 1] | Sulfo-T | 8,3 | 8,9 |
| HS3ST2 [heparan sulfate (glucosamine) 3-O-sulfotransferase 2] | Sulfo-T | 3,2 | 5,9 |

**Supplemental Table 2C. List of genes not significantly different between multiple myeloma and normal donors.**

| **Sub.cate-gory** | **Common.name** | **mean_log2.MM** | **mean_log2.ND** |
| --- | --- | --- | --- |
|  | ARSD (Arylsulfatase D precursor | 2,455898 | 3,545632 |
| Arylsufatases | ARSB (Arylsulfatase B precursor ) | 7,792673 | 6,016552 |
| Fucosyl-T | FUT4 ( fucosyltransferase 4) | 3,256759 | 4,524701 |
| GalNAc-T | B4GALNT4 (beta-1 4-N-acetyl-galactosaminyl transferase 4) | 3,873327 | 4,803736 |
| GalNAc-T | GALNACT-2 (CSGalNAcT2/ChGalNAcT2;chondroitin beta1 4) | 10,39968 | 12,19679 |
| GalNAc-T | GALNT6 (polypeptide N-acetylgalactosaminyltransferase 6) | 2,715328 | 4,259323 |
| GalNAc-T | GALNT7 ( polypeptide N-acetylgalactosaminyltransferase 7) | 5,373482 | 3,179126 |
| GalNAc-T | ChGn (CSGalNAcT1/ChGalNAcT1; chondroitin beta1 4) | 10,72085 | 12,77951 |
| GalNAc-T | GALNT1 (ppGalNAc T1; polypeptide N-acetylgalactosaminyltransferase 1) | 10,38093 | 8,604753 |
| GalNAc-T | GALNT3 (polypeptide N-acetylgalactosaminyltransferase 3) | 9,104833 | 6,854744 |
| GalNAc-T | GALNT2 (polypeptide N-acetylgalactosaminyltransferase 2) | 6,784275 | 8,588264 |
| Gal-T | UGT8 [UDP glycosyltransferase 8 (UDP-galactose ceramide galactosyltransferase)] | 6,477574 | 2,680781 |
| Gal-T | B4GALT1 (UDP-Gal:betaGlcNAc beta 1,4- galactosyltransferase, polypeptide 1) | 10,01647 | 12,13554 |
| Gal-T | CHSY-2 (chondroitin sulfate synthase 3) | 8,310858 | 4,482937 |
| Gal-T | C1GALT1 (core 1 synthase ) | 7,506624 | 5,771384 |
| Gal-T | B4GALT3 (UDP-Gal:betaGlcNAc beta 1,4- galactosyltransferase, polypeptide 3) | 11,92244 | 11,17221 |
| Gal-T | CHSY1 (carbohydrate (chondroitin) synthase 1) | 12,14458 | 11,30576 |
| Gal-T | B3GALNT2 (beta-1 3-N-acetylgalactosaminyltransferase 2) | 10,11754 | 9,475751 |
| Gal-T/GalNAc-T | ABO (transferase A, alpha 1-3-N-acetylgalactosaminyltransferase; transferase B, alpha 1-3-galactosyltransferase). | 5,68094 | 6,298393 |
| GlcNAc-T | B3GNT7 [UDP-GlcNAc:betaGal beta-1,3-N-acetylglucosaminyltransferase 7] | 2,36153 | 3,437423 |
| GlcNAc-T | PIGC [phosphatidylinositol glycan anchor biosynthesis, class C] | 8,139334 | 5,474666 |
| GlcNAc-T | OGT [O-linked N-acetylglucosamine (GlcNAc) transferase (UDP-N-acetylglucosamine:polypeptide-N-acetylglucosaminyl transferase).] | 6,666564 | 10,41657 |
| GlcNAc-T | EXTL2 (multiple exostoses-like 2) | 7,204883 | 4,817901 |
| GlcNAC-T | DPAGT1 [dolichyl-phosphate (UDP-N-acetylglucosamine) N-acetylglucosaminephosphotransferase 1 (GlcNAc-1-P transferase)] | 10,63078 | 9,933726 |
| GlcNAc-T | MGAT1 (mannosyl (alpha-1,3-)-glycoprotein beta-1,2-N-acetylglucosaminyltransferase) | 11,82048 | 11,08882 |
| Glc-T | ALG8 [asparagine-linked glycosylation 8 homolog (S. cerevisiae, alpha-1,3-glucosyltransferase)] | 11,83761 | 9,941341 |
| Glc-T | UGCG [ceramide glucosyltransferase] | 6,66861 | 4,113467 |
| Glc-T | ALG6 [asparagine-linked glycosylation 6 homolog (S. cerevisiae, alpha-1,3-glucosyltransferase)] | 9,564429 | 8,089358 |
| Glc-T | ALG10 [asparagine-linked glycosylation 10 homolog (yeast, alpha-1,2-glucosyltransferase)] | 6,887149 | 5,244151 |
| GlcUA-T | B3GAT3 [beta-1,3-glucuronyltransferase 3 (glucuronosyltransferase I)] | 6,40822 | 5,587793 |
| Heparanases | HPSE (Heparanase) | 5,092053 | 3,085431 |
| Hexosaminidase | HEXA [hexosaminidase A (alpha polypeptide)] | 10,51007 | 9,380491 |
| Hexosaminidase | HEXB (hexosaminidase B preproprotein) | 13,26891 | 12,46433 |
| HS GlcNAc/GlcA Transferase | EXT2 HS Copolymerase [GAG Enzyme] | 9,40597 | 6,803176 |
| Hyaluronoglucosaminidases | HYAL2 (hyaluronoglucosaminidase 2) | 4,954942 | 3,422281 |
| Lysozomal Enzymes/Proteins | LAMP3 (lysosomal-associated membrane protein 3) | 11,55281 | 8,959834 |
| Lysozomal Enzymes/Proteins | LAMP2 (lysosomal-associated membrane protein 2) | 6,602876 | 5,335646 |
| Mannosidase | MAN2A1 (mannosidase, alpha, class 2A, member 1) | 11,46675 | 8,46967 |
| Mannosidase | MAN1B1 [Mannosidase, alpha, class 1B, member 1] | 8,048355 | 6,01235 |
| Mannosidases | NPL (N-acetylneuraminate pyruvate lyase) | 2,753719 | 4,459796 |
| Man-T | PIGM phosphatidylinositol glycan anchor biosynthesis | 8,072242 | 4,282102 |
| Man-T | ALG3 | 10,97327 | 9,658912 |
| Man-T | ALG1 | 8,701346 | 7,475935 |
| Man-T | PIG B | 8,01093 | 6,869728 |
| Man-T | ALG09 | 8,68359 | 7,534395 |
| Miscellaneous | SLC17A5 [solute carrier 17 (anion/sugar)] | 6,642389 | 5,235838 |
| N-glycans-transferase | RPN1 (ribophorin I) | 13,09335 | 11,5564 |
| N-glycans-transferase | DAD 1 | 12,61919 | 11,93272 |
| Nuc. Sugars Transporters | SLC35B4 [solute carrier family 35 member B4] | 8,565539 | 5,212439 |
| Nuc. Sugars Transporters | SLC35D1 [solute carrier family 35 (UDP-glucuronic acid/UDP-N-acetylgalactosamine dual transporter), member D1] | 8,37998 | 5,452653 |
| Nuc. Sugars Transporters | SLC35B2 [solute carrier family 35 member B2] | 9,103638 | 7,298951 |
| Nuc. Sugars Transporters | SLC35A1 [solute carrier family 35 (CMP-sialic acid transporter), member A1] | 11,03594 | 9,890995 |
| Nuc. Sugars Transporters | SLC35A4 [solute carrier family 35, member A4] | 9,194067 | 8,284018 |
| Nuc. Sugars Transporters | SLC35A5 [solute carrier family 35 member A5] | 9,853323 | 8,857359 |
| Nuc. Sugars Transporters | SLC35A3 [solute carrier family 35 (UDP-N-acetylglucosamine (UDP-GlcNAc) transporter), member A3] | 5,482542 | 4,326242 |
| Nuc. Sugars Transporters | SLC35E3 [solute carrier family 35 member E2] | 9,090375 | 8,205689 |
| Nucleotide Synthesis | GALK1 [galactokinase 1] | 2,529805 | 4,848844 |
| Nucleotide Synthesis | GALK2 [galactokinase 2] | 7,824943 | 5,447528 |
| Nucleotide Synthesis | GPI [glucose phosphate isomerase] | 10,86966 | 8,969666 |
| Nucleotide Synthesis | PAPSS1 [3'-phosphoadenosine 5'-phosphosulfate synthase 1] | 11,70271 | 10,31002 |
| Nucleotide Synthesis | PGM1 [phosphoglucomutase 1] | 10,81727 | 9,480807 |
| Nucleotide Synthesis | GNB1 [guanine nucleotide binding protein beta-1] | 11,37405 | 9,897777 |
| Nucleotide Synthesis | TSTA3 [tissue specific transplantation antigen P35B] | 9,092785 | 8,026495 |
| Nucleotide Synthesis | GMPPA [GDP-mannose pyrophosphorylase A] | 7,184008 | 8,395761 |
| Nucleotide Synthesis | CMAS [cytidine monophosphate N-acetylneuraminic acid synthetase] | 7,51632 | 6,37141 |
| Nucleotide Synthesis | PMM2 [phosphomannomutase 2] | 9,459207 | 10,13784 |
| Nucleotide Synthesis | KHK [ketohexokinase] | 7,031294 | 6,109176 |
| Nucleotide Synthesis | HK3 [hexokinase 3 (white cell)] | 2,706194 | 3,679003 |
| Nucleotide Synthesis | UGDH [UDP-glucose dehydrogenase] | 10,33297 | 9,507177 |
| Sia-T | ST3Gal5 [ST3 beta-galactoside alpha-2,3-sialyltransferase 5] | 11,04039 | 9,167709 |
| Sia-T | ST3Gal6 [ST3 beta-galactoside alpha-2,3-sialyltransferase 6] | 11,77414 | 8,380247 |
| Sia-T | ST8SIA4 [ST8 alpha-N-acetyl-neuraminide alpha-2,8-sialyltransferase 4] | 9,795252 | 7,188241 |
| Sia-T | ST6GalNAc6 [ST6 (alpha-N-acetyl-neuraminyl-2,3-beta-galactosyl-1,3)-N-acetylgalactosaminide alpha-2,6-sialyltransferase 6] | 5,458059 | 5,0899 |
| Sulfatases | GALNS (N-acetylgalactosamine-6-sulfatase precursor) | 6,095176 | 6,825654 |
| Sulfo-T | CHST11 [carbohydrate (chondroitin 4) sulfotransferase 11] | 8,767717 | 6,237763 |
| Sulfo-T | HS3ST2 [heparan sulfate (glucosamine) 3-O-sulfotransferase 2] | 3,259836 | 5,905553 |
| Sulfo-T | HS2ST1 [heparan sulfate 2-O-sulfotransferase 1] | 6,490913 | 4,15651 |
| Sulfo-T | HS6ST1 [heparan sulfate 6-O-sulfotransferase 1] | 8,34356 | 8,952592 |
| Sulfo-T | CHST14 [carbohydrate (N-acetylgalactosamine 4-0) sulfotransferase 14; aka dermatan 4 sulfotransferase 1] | 6,055277 | 5,173179 |
| Sulfo-T | CHST13 [carbohydrate (chondroitin 4) sulfotransferase 13] | 2,326448 | 2,814607 |
