# Supplementary material for: The Glycome of Normal and Malignant Plasma Cells
Source: PLoS One. 2013 Dec 26;8(12):e83719. doi: 10.1371/journal.pone.0083719 (PMC3873332; doi:10.1371/journal.pone.0083719)
Supplement: Table S3 — Overview of glycome genes categorized by gene family and subfamily. (DOC) [file pone.0083719.s004.doc]

**Supplemental Table S3: Overview of glycome genes categorized by gene family and subfamily**

| **Gene Family** | **Gene-SubFamily** | **No of expressed genes in one of the investigated groups** |
| --- | --- | --- |
| **Glycosyltransferases** | Fucosyltransferases | 12 |
|  | Sialyltransferases | 12 |
|  | Sulfotransferases | 20 |
|  | Galactosyltransferases | 16 |
|  | N-acetylgalactoaminetransferases | 18 |
|  | Glucosyltransferases | 9 |
|  | N-acetylglucosaminetransferases | 27 |
|  | GlucUA-transferases | 5 |
|  | Mannosyltransferases | 12 |
|  | HS GlcNAC/GIA transferases | 2 |
|  | Hyaluronansynthases | 1 |
|  | N-Glycantransferases | 4 |
|  | Xylosyltransferases | 2 |
|  |  |  |
| **Glycan degradation (GD)** | Arylsulfatases | 4 |
|  | Fucosidases | 1 |
|  | Galactosidases | 3 |
|  | Heparanases | 1 |
|  | Hexosaminidases | 2 |
|  | Hyaluronoglucosaminidases | 2 |
|  | Iduronidases | 1 |
|  | Lysosomal enzymes | 6 |
|  | Mannosidases | 10 |
|  | Sialidases | 2 |
|  | Sulfatases | 3 |
|  | Sulfohydrolases | 1 |
|  |  |  |
| **Sugar s/t** | Nucleotide sugar transporters | 20 |
|  | Nucleotide synthesis | 34 |
|  |  |  |
| **Phosphatidylinositol glycan biosynthesis** | GPI Biosynthesis | 13 |
|  |  |  |
| **Total** |  | **243** |
