# Supplementary material for: The Glycome of Normal and Malignant Plasma Cells
Source: PLoS One. 2013 Dec 26;8(12):e83719. doi: 10.1371/journal.pone.0083719 (PMC3873332; doi:10.1371/journal.pone.0083719)
Supplement: Table S4 — A. Genes involved in Lasso classifier to distinguish multiple myeloma vs. bone marrow plasma cells (BMPC). Genes relevant for the classification of MM vs BMPC samples are shown in the right column. The relative weighting of each gene for the classifier is shown on the right column. The intercept for this classifier is 6.29. B. Sensitivity/Specificity analysis of Lasso classifier. Results of the classification of samples resulting in best possible sensitivity and high specificity for classification. (DOC) [file pone.0083719.s005.doc]

**Supplemental Table S4A. Genes involved in Lasso classifier to distinguish multiple myeloma vs. bone marrow plasma cells (BMPC)**

| **Genes** | **Weighting** |
| --- | --- |
| GPI | 0,868541 |
| SLC35B4 | 0,553126 |
| UGT8 | 0,35131 |
| PIGM | 0,329798 |
| CHSY3 | 0,28885 |
| ST6GAL1 | 0,22723 |
| SLC35D1 | 0,219981 |
| HYAL2 | 0,2159 |
| B4GALT1 | -0,1286 |
| UGGT2 | -0,14195 |
| GALNT6 | -0,20627 |
| CSGALNACT1 | -0,32659 |
| HS3ST2 | -0,57228 |
| CSGALNACT2 | -0,64301 |
| OGT | -0,85203 |

**Table S4B. Sensitivity/Specificity analysis of Lasso classifier**

| Sensitivity | 1 |
| --- | --- |
| Specificity | 0.90 |
| Prog. Pred Value | 1.0 |
| Neg Pred Value | 0.96 |
