# Supplementary material for: The Glycome of Normal and Malignant Plasma Cells
Source: PLoS One. 2013 Dec 26;8(12):e83719. doi: 10.1371/journal.pone.0083719 (PMC3873332; doi:10.1371/journal.pone.0083719)
Supplement: Table S5 — 38-gene signature correlating with overall survival. Using a penalized Cox proportional hazard regression model we identified a 38-glycan gene signature significantly associated with overall survival (OS; P<0.001, Figure 4). The absolute value of the LASSO coefficient indicates the weight of the respective gene for the gene score. For genes in which upregulation contributed to high risk condition, the PIG-M gene of the GPI-anchor biosynthesis pathway and the CHSY1 gene of the GAG chondroitin sulfate pathway had the highest coefficients. Negative or positive values of the LASSO coefficient indicate if down or upregulation of the respective gene contributed to the high risk profile. For genes in which downregulation contributed most to high risk profile 2 glycan degradation genes were identified. (DOC) [file pone.0083719.s006.doc]

**Supplemental Table S5: 38-gene signature correlating with overall survival**

| **gene** | **coef.lassoOS.** |
| --- | --- |
| PIGM | 0,233547139 |
| CHSY1 | 0,125231055 |
| GCNT1 | 0,123155465 |
| MFNG | 0,10847167 |
| GALNT1 | 0,079571567 |
| SULF2 | 0,074102494 |
| ST8SIA4 | 0,062499374 |
| ST6GAL1 | 0,050120248 |
| GALNT3 | 0,036256313 |
| PAPSS2 | 0,034676985 |
| SLC35C1 | 0,034008906 |
| ALG13 | 0,029485317 |
| HS3ST3B1 | 0,021957683 |
| SLC35E3 | 0,020356455 |
| GALT | 0,015497172 |
| DSEL | 0,010194231 |
| ST3GAL1 | 0,010023182 |
| SLC35B4 | 0,005665426 |
| HPSE | 0,000719033 |
| ALG10 | 0,000202253 |
| GALNT2 | -0,001601979 |
| SLC35D2 | -0,003860946 |
| MAN2A1 | -0,004303488 |
| SLC35F1 | -0,005202267 |
| GM2A | -0,008814708 |
| B3GALNT1 | -0,01180813 |
| HS3ST2 | -0,015364674 |
| UGT2B17 | -0,018238628 |
| CSGALNACT1 | -0,025776267 |
| IDUA | -0,05668129 |
| B3GAT2 | -0,058947716 |
| UGGT2 | -0,061814861 |
| GCNT2 | -0,067287872 |
| GALNT11 | -0,072023193 |
| CHSY3 | -0,076737139 |
| MGAT4A | -0,104019127 |
| GALC | -0,183888855 |
| NEU1 | -0,205066474 |
